# Risk and Protective Factors for Suicidal Thoughts and Behaviors Among Asian American Young Adults: A Systematic Review

**DOI:** 10.3390/healthcare13010018

**Published:** 2024-12-25

**Authors:** Yong Li, Tzu-Fen Chang, Qing Zhou, Kathryn Li, Philip Baiden, Mark S. Kaplan

**Affiliations:** 1 Orien Levy Woolf Division of Social Work, Texas Woman’s University, Denton, TX 76204, USA; 2 Department of Human Development and Child, Adolescent, and Family Studies, California State University, Bakersfield, CA 93311, USA; tchang1@csub.edu; 3 Department of Psychology, University of California, Berkeley, CA 94720, USA; qingzhou@berkeley.edu (Q.Z.); kathryn.li@berkeley.edu (K.L.); 4 School of Social Work, University of Texas at Arlington, Arlington, TX 76019, USA; philip.baiden@uta.edu; 5 Luskin School of Public Affairs, University of California, Los Angeles, CA 90095, USA; kaplanm@luskin.ucla.edu

**Keywords:** suicidal thoughts and behaviors, Asian American, young adults, risk factors, protective factors

## Abstract

**Background**: Asian American (AA) young adults, including AA college students, may experience more suicidal thoughts and behaviors (STBs) compared to other racial and ethnic groups of the same age. To the best of our knowledge, this study is the first systematic review of the risk and protective factors for STBs with a focus on AA young adults. **Methods**: Informed by the social-ecological perspective and the cultural model and theory of suicide, this study systematically reviews the risk and protective factors for STBs among AA young adults. Based on 22 research articles published between 1998 and 2023, we analyzed and discussed the effects of 37 risk and 15 protective factors at the individual, relationship, community, societal, and cultural levels. **Results**: Most risk factors are at the individual level (e.g., depressive symptoms and hopelessness), followed by factors at the cultural level (e.g., acculturation and acculturative stress), the relationship level (e.g., family problems and romantic relationship problems), the community level (e.g., verbal threats on campus), and the societal level (e.g., public stigma about mental health). Also, most protective factors are at the individual level (e.g., self-reliance and fear of suicide), followed by the relationship level (e.g., social support and family responsibilities), the community level (e.g., religious affiliations), and the cultural level (desire not to burden others). **Conclusions**: This systematic review emphasizes the need for future research to explore cultural factors, subgroup differences, and longitudinal designs, while advocating for culturally specific prevention and intervention strategies to improve mental health outcomes for AAYAs.

## 1. Risk and Protective Factors of Suicide

Young adults aged 18–26 are faced with unique developmental, social, and economic challenges, yet they are often categorized as either adolescents or adults in policy, program development, and research [1]. This underscores the need for more targeted efforts focused specifically on young adults. Suicidal thoughts and behaviors (STBs), including suicidal ideations, plans, attempts, and deaths by suicide, have been increasing in the United States for the past few decades [2]. Research has shown STBs may disproportionately affect Asian Americans (AAs), especially AA young adults (AAYAs). From 2011 to 2020, the suicide rate among AAYAs (aged 18–26) increased by 75%, which was higher than their White (17%), Hispanic (63%), and American Indian (56%) counterparts [3]. Within the AA community, data showed that AAYAs aged 18–25 experienced higher levels of STBs compared to Asians in other age groups [4]. In addition, AA college students were more likely to have suicidal thoughts [5,6,7] and suicide attempts [7] than students of other racial and ethnic backgrounds (e.g., White, black, and Hispanic). AA college students were the second largest racial group in campus suicides, making up 10% of such deaths, with White students being the largest group represented [8].

There has been extensive research examining the risk and protective factors for STBs. Risk factors are factors that are associated with a heightened likelihood of developing STBs. In contrast, protective (or asset) factors include factors that are associated with decreased likelihood of STBs or factors that interact with the risk factor by mitigating the impact of risk factors on STB outcomes [9]. To our knowledge, there has been no systematic review focusing specifically on risk and protective factors for STBs among AAYAs, although there have been such reviews on AA adolescents [10,11]. Our study aimed to fill this gap by conducting a systematic review of the protective and risk factors for STBs among AAYAs.

Given the complexity of understanding the risk and protective factors of STBs, a theoretical framework is valuable for organizing these factors and studying the mechanisms of suicide. One such framework is the Social-Ecological Suicide Prevention Model (SESPM), which includes four levels of risk and protective factors for suicide: individual, relationships, community, and societal levels [12]. Rooted in the social-ecological perspective [13], the SESPM stresses that suicide is a complex phenomenon that is influenced by multi-dimensional and interconnected factors [12]. Informed by the SESPM, Cramer and Kapusta [14] compiled a comprehensive list of risk and protective factors for STBs across the individual, relationship, societal, and community levels. Specifically, individual factors include biological (e.g., sex), socio-demographic (e.g., gender), psychiatric (e.g., personality disorders), and psychological (e.g., hopelessness) factors. The relationship level examines the influence of close relationships on suicidal behavior (e.g., family and romantic relationships). At the community level, the focus shifts to broader community influences (e.g., community centers and schools). The societal level considers larger societal factors that can affect suicide rates (e.g., structural discrimination and racism).

To better understand suicide among specific cultural or minority groups, Chu et al. [15] proposed the Cultural Theory and Model of Suicide (CTMS). The CTMS emphasizes the role of cultural factors such as cultural sanctions, idioms of distress, cultural stress, and social discord [15]. Specifically, cultural sanctions are the norms or beliefs within a culture that decide if suicide is seen as acceptable or unacceptable. Idioms of distress are the specific ways people from different cultures show STBs. Cultural stress includes the challenges faced by people from minority backgrounds due to their social identity (e.g., discrimination). Social discord refers to social issues like family conflict, lack of community connection, and feeling isolated from family or friends. This framework has been applied to Asian Americans and other minority groups, with some empirical evidence supporting its application [16,17].

## 2. The Present Review

Drawing upon the SESPM and the CTMS frameworks, we conducted a systematic review of the risk and protective factors for STBs among AAYAs. Specifically, we reviewed the risk and protective factors for STBs at the individual, relationship, community, societal, and cultural levels for AAYAs.

## 3. Review Method

### 3.1. Data Sources and Selection Procedure

We used the Preferred Reporting Items for Systematic Reviews and Meta-Analyses (PRISMA) method for our systematic review [18]. The PRISMA method is appropriate for non-meta-analysis, systematic review studies. Electronic searches were undertaken using the following five databases: PsycINFO, CINAHL, Pubmed, Web of Science, and ERIC. We limited our literature search to articles published between 1998 and 2023. Search keywords included three sets:

(i). (Asia* or Japan* or Korea* or Chinese* or Bangladesh* or Bhutanese* or Burmese* or Cambodia* or Filipin* or Phillipin* or Pilipin* or Hmong* or Nepal* or Pakistan* or Singapore* or Sri Lanka* or Taiwan* or Thai* or Vietnam* or Afghani* or Asian Indian* or Indones* or Lao* or Malays* or Mongol* or south Asia* or southeast Asia* or east Asia* or central Asia* or west Asia*) and America*;
(ii). College students, university students, young adults, or young people;
(iii). suicide*.

**Figure 1:**
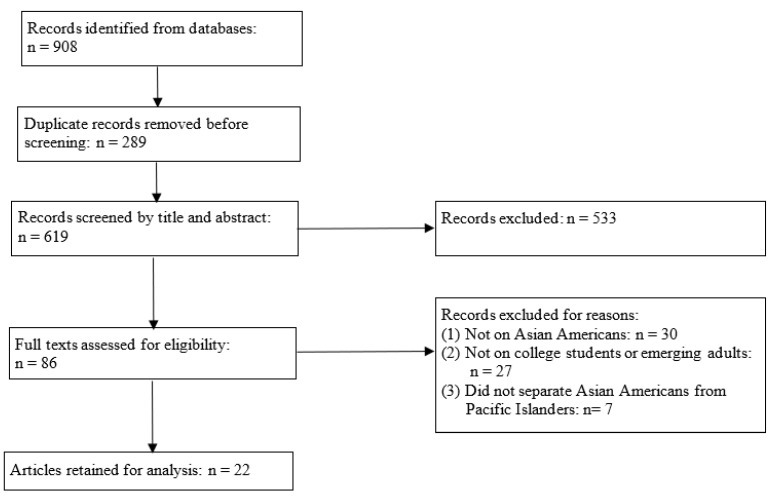
Process and Results of Article Selection.

Using these keywords ensured that we were able to identify articles focusing on Asian Americans as a whole or specific AA subgroups, college students and/or young adults, and different STBs. In our search results, we excluded studies that were not written in English, as well as some special types of articles, including dissertations and theses, commentaries, editorials, and letters. The preliminary search yielded 908 articles, all of which were downloaded to a Zotero library. An initial review based on the title prompted us to remove 289 duplicates, resulting in 619 articles. Then, we reviewed the abstract of the 619 articles. In this process, we excluded articles that did not examine STBs among U.S. populations. This resulted in the deletion of 533 articles. The next step involved examining the full text of the remaining 86 articles. Another 64 articles were removed because they did not focus on STBs among AAYAs. As a result, a total of 22 articles were included in our synthesis (see Figure 1). Because two studies [19,20] included more than one sample, the total number of samples in our analysis was 25.

### 3.2. Data Extraction and Profile

**Figure 2:**
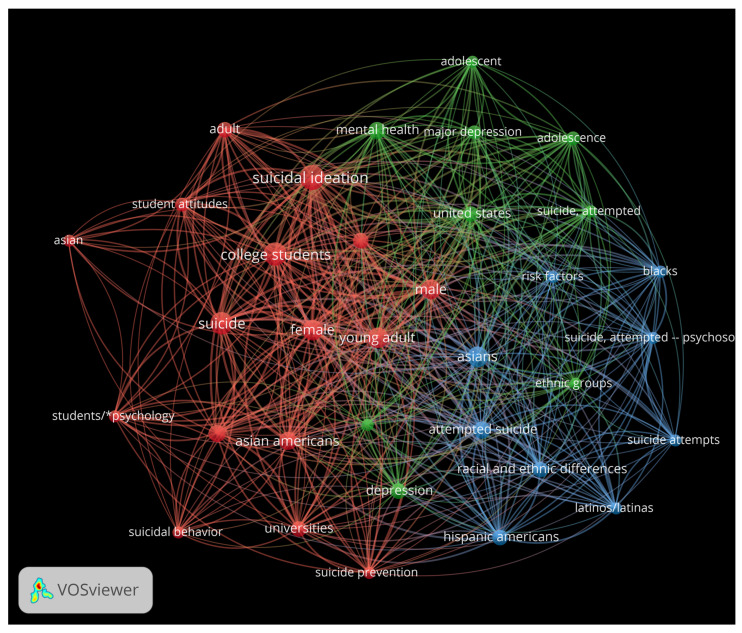
Network visualization of keywords extracted from the 22 selected studies.

To describe the data, we began by using VOSviewer (version 1.6.20), a free tool for constructing and visualizing bibliometric networks, to conduct a keyword co-occurrence analysis. We input the bibliographic information from the 22 selected studies into VOSviewer. The minimum number of occurrences for a keyword was set to 3, resulting in 37 keywords. We removed three keywords: human, humans, and descriptive statistics, as they are not relevant to the purpose of our study. Figure 2 illustrates the network visualization of these keywords. As shown in Figure 2, some of the keywords with the highest total link strength include “female”, “young adult”, “suicide”, “suicidal ideation”, “mental health”, “major depression”, “college students”, “Asians”, “United States”, and “racial and ethnic differences”.

For a more thorough description of the data profile, below is a summary of the characteristics of the 22 selected studies and 25 selected samples, including race, ethnicity, sampling method, sample size, generation status, research methods, and suicide outcomes (see Table 1, Table 2, Table 3, Table 4 and Table 5).

Race and Ethnicity. In Table 1, 12 studies (55%) focused on AAs, whereas 10 studies (45%) recruited AAs and other racial groups. Among the 12 studies with only AAs, two studies conducted subgroup analysis to understand heterogeneities within AAs. Specifically, Lane et al.’s [19] study included Bangladeshi, Asian Indian, and Pakistani college students, while Park’s [20] study included Chinese and Filipino young adults. Among the 10 studies that included diverse racial groups, seven (70%) included European Americans, eight (80%) included African Americans, nine (90%) included Latinos, four (40%) included biracial or multiracial samples, three (30%) included Native Americans, one (10%) included other Americans, and one (10%) included Arab Americans. In addition, six studies (60%) reported results separately for Aas, and four (40%) treated race as a dummy variable, and results were reported based on the whole sample.

Other Sample Characteristics. Table 2 details additional sample characteristics. Specifically, five samples (20%) had fewer than 50 participants, six samples (24%) had between 51 and 100, six samples (24%) had between 101 and 500, two samples (8%) had between 501 and 1000, and six samples (24%) had more than 1000. Four samples (16%) focused on non-college-attending young adults, while 21 (84%) targeted college students. In terms of sex, 14 samples (56%) had mostly female participants, five (20%) had an even male-to-female ratio, three (12%) had mostly male participants, and three (12%) did not report sex. Most samples (76%) did not specify generational status. Of those that did, two samples (8%) had an even generational distribution, two (8%) were mostly first generation and two (8%) were mostly second generation or beyond. Similarly, socioeconomic status was not reported for the majority of samples (92%); only two samples (8%) included this information, indicating a middle-class status and moderate financial stress among participants.

Sampling Methods. Table 3 shows information on the region and duration of data collection, the method of data collection and analysis, and the sampling method of the 22 reviewed studies. Most studies utilized national data (n = 12; 55%) rather than regional data and cross-sectional data (n = 19; 86%) rather than longitudinal data. Most studies (n = 18; 82%) adopted a quantitative approach to data collection and analysis rather than qualitative or mixed methods approaches. In terms of sampling methods, 14 studies (64%) used a convenience sampling method, six studies (27%) used a random sampling method, and two studies (9%) used both random and purposive sampling methods. The last sampling procedure involved using random sampling at the beginning and then purposive sampling to deliberately sample participants who had exhibited suicidal ideation or attempted suicide within the past 12 months.

Suicide Outcomes Assessed. As shown in Table 4, 13 out of the 22 (59%) reviewed studies examined recent suicidal ideation as the outcome variable. Only one study (5%) examined lifetime suicidal ideation. Regarding suicide attempts, two studies (9%) examined recent suicide attempts and three (14%) studied lifetime suicide attempts. Six studies (28%) included a composite measure of both suicidal ideation and suicide attempts. Among these six studies, one study (5%) focused on recent suicidality, and one (5%) on past suicidality (during college). Focusing on both recent and lifetime suicidality measures, the remaining four studies (18%) used a comprehensive measure to assess the frequency and intensity of suicidal ideation and the frequency, threat, and future likelihood of suicide attempts. In addition, 11 studies (50%) assessed suicide using dichotomous measures, and 11 studies utilized continuous measures. The most frequently used continuous measure was the Suicide Behaviors Questionnaire-Revised [40].

### 3.3. Risk and Protective Factors for Suicide

Based on the 22 reviewed articles, the risk and protective factors for suicide outcomes among AAYAs are summarized below (see Table 5). Guided by the SESPM [14], a suicide model rooted in the social-ecological perspective, we categorized risk and protective factors into four levels: individual, relationship, community, and societal. Integrating a cultural framework on STBs for minorities (i.e., the CTMS), we also included a cultural dimension for both the risk and protective factors.

### 3.4. Risk Factors

Individual level. Health (including mental and behavioral health) has been identified as a potential risk factor for suicide outcomes among AAYAs. Health issues like weight-related problems and sleep disturbances have been linked to STBs among AA college students [38]. Mental health factors include depression or depressive symptoms [19,20,26,30,34], anxiety [34], and mental health medication use [28]. However, some studies, such as Hirsch et al. [33], found no significant risk associated with depression in small samples (n = 21), and Wong et al. [28] suggested that medication use should be considered a proxy measure for mental health problems, rather than a risk factor directly related to suicide. Additionally, behavioral health factors like computer recreation and chemical use have also been linked to STBs among AAYAs [26].

Four psychological factors have been identified as potential risk factors for STBs among AA college students: hopelessness [19,36], loneliness [26], low self-esteem [30], and generic state shame [30]. However, Hirsch et al. [33] did not find a significant association between hopelessness and STBs, which may be attributed to the study’s small sample size. In addition, personal traits and characteristics have also been identified as individual-level risk factors for STBs among AA college students. These include high levels of perfectionism [6], low self-worth [22,29], inadequate coping skills [22], and poor social problem-solving skills [6].

Studies informed by the Interpersonal Theory of Suicide [41] have explored perceived burdensomeness and thwarted belongingness in relation to STBs among AAYAs. Perceived burdensomeness was linked to suicidal ideation in multiple studies on AA college students [29,35], while thwarted belongingness showed mixed results among AA young adults, including college students [30,35]. These findings underscore the need for further research to confirm the theory’s applicability to this group.

Academic performance is another key factor, with poor performance correlating with STBs [24,28,38], though high performance can also coincide with STBs due to pressure from parental expectations [22,29]. Demographic factors also play a role in shaping AAYAs’ STBs. AA female college students report more lifetime suicidal ideation but not more attempts compared to males [38], sexual minority students report higher lifetime suicidal ideation [24], and financial problems and food insecurity are associated with recent suicidal ideation [26,28]. However, AA female college students did not report more suicide attempts than their male counterparts [38].

Relationship Level. Several family and relationship factors have been identified as risk factors for suicidal outcomes among AAYAs: conflict with family members [37], family problems [28], poor-quality romantic relationships [22], conflict with romantic partners [37], peer and friend conflicts [37], and interpersonal shame [30]. However, the specific issues within family problems and the different types of peer and romantic relationship conflicts remain underexplored and not fully detailed in the studies.

Community Level. Verbal threats on campus have been linked to higher rates of suicidal ideation and attempts among AA college students [24]. Additionally, involvement in student organizations has been associated with passive suicidal ideation and serious consideration of suicide, potentially due to increased exposure to racism and reduced participation in other social groups [28]. However, more research is needed to explore factors that mediate or moderate these relationships.

Societal Level. Goodwill and Zhou [32] reported that public stigma about mental health and treatment was a risk factor for STBs based on a large national sample of college students, including AA students. Since they did not report results separately for AA students [32], future research should consider investigating the interaction effects between race and public stigma to examine whether the effect of public stigma varies across racial groups.

Cultural/Language/Race/Ethnicity Level. Consistent with the CTMS [15], nine cultural risk factors for suicide outcomes among AAYAs have been identified: language-based acculturation stress [25], cultural conflict [29], familial acculturative stress [31], family shame [30], racial and ethnic discrimination [26,29,31,34], gender discrimination [29], gendered racial microaggressions [23], online racism [35], and internalized racism [23]. It is important to note that although these factors are derived from the CTMS, they have not been examined through cross-cultural studies and, therefore, only represent culture-related elements tied to race, ethnicity, gender, and acculturation.

### 3.5. Protective Factors

Individual level. Our review identified eight individual-level protective factors for suicide outcomes among AA college students: personal reasons for living [21,27,34], self-reliance [27], fear of suicide [27], optimism [39], sufficient sleep [38], physical activity [38], and both independent and interdependent self-construals [29]. However, further research is needed to explore how these factors may interact in shaping STBs among AAYAs.

Relationship Level. Our review identified four relationship-level protective factors for AA college students’ suicide outcomes: social support [27], socializing [26], family responsibilities [26] and living with family or having a roommate [28].

Community Level. At this level, religious affiliations and the transition to college life were identified as protective factors. AA college students with religious affiliations were found to have a lower risk of experiencing suicidal thoughts in the past 12 months [28]. Additionally, Chung [22] found that the college transition served as a protective factor for long-term suicidal ideation and attempts among AA female college students, as it provided them with increased autonomy from parental control.

Societal Level. Cramer and Kapusta [14] identified several protective factors at the societal level, such as the economy and geographic locations. However, our review did not reveal any protective factor at this level among AAYAs.

Cultural Level. AAs often view the self as interconnected with others rather than as an independent entity [42]. Supporting this, Tran et al. [27] found that some AA college students did not report recent suicidal ideation when they expressed a desire not to burden family and friends.

### 3.6. Methodological Bias in Data

The 22 studies reviewed revealed several methodological biases: (a) Self-selection recruitment methods may skew results towards individuals with STBs; (b) Self-administered surveys can lead to inaccurate reporting of STBs; (c) Cross-sectional designs limit causal inferences; (d) Many studies analyzed AAs as a panethnic group, ignoring subgroup diversity; (e) Sampling from specific contexts, like psychology classes, may limit sample diversity; (f) Small sample sizes affect generalizability; (g) Regional data may not apply universally; (h) Yes/no questions may miss the severity of suicidality; (i) Solely using suicidal ideation may not capture the full scope of suicidality; (j) Generational and socioeconomic factors were often not assessed; (k) Retrospective recall can introduce bias by through inaccurate memories, potentially compromising the reliability of the data; (l) Some studies did not report separate results for Asian Americans but instead presented findings by comparing Asian Americans to Whites e.g., [38].

Overall, the certainty of the evidence in this review is moderate, as findings were consistent across studies but were often limited by small sample sizes, cross-sectional designs, and reliance on self-reported measures. Meanwhile, the potential for reporting bias in this review is moderate, as most included studies focused on significant findings and provided limited discussion of inconclusive results, which may indicate an underrepresentation of nonsignificant outcomes in the literature.

## 4. Discussion

Our systematic review of risk and protective factors for STBs is the first to specifically focus on AAYAs. Informed by the SESPM, a theoretical framework rooted in the social-ecological perspective, and the CTMS, a cultural framework on suicide, our study highlighted the role of these factors at the individual, relationship, community, societal, and cultural levels among this population (see Table 5).

Our review highlighted that many risk and protective factors were reported in only one or two studies (see Table 5). Sometimes, conflicting findings are reported, underscoring the need for more focused research on these aspects for AAYAs. As we reviewed previously, examples include academic performance, perceived burdensomeness, thwarted belongingness, and the transition to college. For example, while one study suggested that the college transition might reduce STBs by increasing autonomy for AA college students [22], other research indicates it can negatively affect mental health in the general college population [43].

Cramer and Kapusta’s [14] review of the risk and protective factors for suicide among the general population covered 79 risk factors and 47 protective factors, far exceeding our list of 37 risk and 15 protective factors. When compared to their study, our study identified 17 common risk factors (e.g., depressive symptoms, substance use) and six common protective factors (e.g., personal reasons for living, optimism). Therefore, future research on STBs among AAYAs should address additional risk factors (e.g., preparatory behaviors and exposure to suicide) and protective factors (e.g., life satisfaction, school-based support, firearm laws, and mental health funding) that are reported in Cramer and Kapusta’s [14] review.

Additionally, our review identified 20 unique risk factors and nine unique protective factors (see Table 5), confirming the need for more population-specific studies, as emphasized by Cramer and Kapusta [14]. These additional factors reflect the unique demographic and cultural characteristics of our study population. For example, we identified five individual-level risk factors—loneliness, excessive computer use, academic performance, weight problems, and food insecurity—that were not noted by Cramer and Kapusta [14]. We also found five individual-level protective factors unique to our population: self-reliance, sufficient sleep, physical activity, independent self-construal, and interdependent self-construal. Notably, Cramer and Kapusta’s [14] study lacked nine cultural-level factors, highlighting the crucial role of cultural factors and the need for further research in this area. Our review indicates that cultural factors are under-researched in general population studies [2,14] as well as in research on AAs [10,11] and college students [43,44].

It is worth noting that cultural factors identified in our study span across individual, relationship, community, and societal levels, highlighting the interplay between the SESPM and CTMS frameworks. At the individual level, cultural conflict and acculturative stress negatively impact mental health and elevate STB risks [45,46]. Family shame, rooted in AA family dynamics, acts as a relationship-level cultural risk factor [30]. At the societal level, discrimination and racism contribute to STBs among AAYAs [26,35]. Although no community-level factors were identified, concepts such as cultural integrity on college campuses and inclusive campus climates have been linked to better mental health outcomes for sexually minority students [47,48]. This suggests the need for future research on campus culture and STBs among AAYAs.

Cultural factors can interact with risk factors at other levels and contribute to STBs among AAYAs. For example, personal traits like perfectionism and low self-worth may be exacerbated by cultural dynamics within AA families, where high parental expectations and critical attitudes are common [22]. Additionally, the Asian cultural tendency to discourage direct emotional expression can hinder AAYAs’ development of effective emotional support-seeking skills, leading to poor coping mechanisms [49]. Future research should explore the interplay between cultural risk factors and risk factors at other levels. Qualitative approaches can help reveal how various factors interact across different levels to shape suicide outcomes among AAYAs.

Our study also found a lack of subgroup analysis within the AA community in the existing literature. Of the 22 reviewed articles, only two included such analyses: Lane et al. [19] found that hopelessness was associated with suicidal ideation among Bangladeshis and Asian Indians, but not Pakistanis, with a stronger link for Asian Indians. Park [20] observed higher depression levels among Filipino Americans compared to Chinese Americans but no significant difference in suicidal ideation or the relationship between depression and suicidal ideation. Future research should include more subgroup analyses to better understand how sociocultural factors affect suicide outcomes across different AA ethnic groups. Recent updates to race/ethnicity coding by the U.S. Census Bureau and the CDC will provide access to more detailed and disaggregated data, which can support this line of inquiry and improve the accuracy of subgroup analyses.

Our review highlights a shortage of studies focusing specifically on AA young adults outside of the college environment. Eighteen of the 22 reviewed studies were conducted with AA college students, who may face different challenges and have access to resources not available to their non-college-attending peers. The structured support systems and unique stressors of college life may not reflect the broader experiences of all Asian American young adults. Therefore, findings from college student samples may not fully represent the diverse experiences of the entire AA young adult population.

Finally, longitudinal studies on STBs among AAYAs are still lacking. A risk or protective factor cannot inform prevention and intervention efforts unless it is a causal factor [2]. Most of the research that we reviewed is correlational, which means that the factors are, at best, correlates for suicide, not causal risk or protective factors. Future research should consider longitudinal designs to better inform mental health practice that aims to reduce suicide among AAYAs.

### Limitations

While this systematic review provides valuable insights into the risk and protective factors for suicidal thoughts and behaviors among Asian American young adults, several limitations should be acknowledged. First, the review did not include a meta-analysis due to the heterogeneity of the included studies in terms of their methodologies, populations, and measured outcomes. The lack of meta-analysis limits our ability to quantitatively synthesize the findings and assess the overall effect sizes of the identified factors. Second, while this review provides insights into STBs among AAYAs, it is important to note a significant gap in the literature: no studies were identified that included data on AAYAs who died by suicide. This omission limits our understanding of the full spectrum of STBs within this population, particularly given existing evidence in broader research that differences often exist between individuals with suicidal ideation, attempts, and deaths [50]. Future research is needed to specifically investigate suicide deaths among AAYAs to better understand the sociocultural and systemic factors influencing this outcome and to inform more comprehensive prevention strategies. Third, the review primarily focused on college students, which may not fully represent the experiences of all Asian American young adults, particularly those who are not attending college. Finally, the age range we focused on (18–26 years) is somewhat arbitrary. While the term “young adults” is commonly used in the literature, it may not adequately capture the rapid growth and developmental changes characteristic of this stage. We found that existing systematic reviews defined “young adults” using varying age ranges. For instance, Li et al. [51] studied young adults aged 18–24, Goodwin et al. [52] included individuals up to age 25, and Aguey-Zinsou et al. [53] extended the age range to include young adults up to 30 years. Still, in medical research, the term is sometimes used to include people aged 15 to 49 [54]. Future research should consider adopting the term “emerging adults” instead of “young adults”. This term, grounded in a more theoretically sound framework [55], captures the unique developmental tasks and psychosocial experiences of individuals aged 18–29.

## 5. Conclusions

This systematic review underscores the critical need for tailored research and interventions addressing STBs among AAYAs. The findings highlight significant gaps, particularly in cultural factors and subgroup differences within the AA population, which necessitate further investigation. Future research should also prioritize longitudinal studies to establish causal relationships between risk and protective factors and STBs. In terms of practice implications, mental health professionals and campus support services should integrate culturally specific approaches and address unique stressors such as acculturative stress and AA family dynamics. By addressing these areas, we can better develop and implement targeted prevention and intervention strategies, ultimately improving mental health outcomes for AAYAs.

## Figures and Tables

**Table 1:** Race Profile of the 22 Reviewed Studies.

	*n* (% ^a^)	Article
Focused Race(s) in 22 Reviewed Studies		
Only Asian (Including International Asians)	12 (55%)	[19,20,21,22,23,24,25,26,27,28,29,30]
Asian Americans and Other Racial Groups	10 (45%)	[6,31,32,33,34,35,36,37,38,39]
Results Reported Separately for Asian Americans (correlation or regression) ^b^	6 (60%)	[31,33,34,35,36,39]
Results Not Reported Separately (Race Entered As a Dummy Variable for Whole Sample) ^b^	4 (40%)	[6,32,37,38]
Asian Americans and Other Racial Groups (n = 10)		
European Americans ^b^	7 (70%)	[6,31,32,33,36,37,38]
African Americans ^b^	8 (80%)	[31,32,33,35,36,37,38,39]
Latinos ^b^	9 (90%)	[31,32,33,34,35,36,37,38,39]
Native Americans (American Indian, Alaskan Native, or Native Hawaiian/Pacific Islander) ^b^	3 (30%)	[31,32,35]
Biracial/Multiracial ^b^	4 (40%)	[31,32,35,37]
Arab Americans ^b^	1 (10%)	[32]
Others ^b,c^	1 (10%)	[33]

Note. Numbers used to denote each article correspond to the number in the reference list. ^a^ The sum of the percentages was above 100% because many studies had more than one other racial group. ^b^ Percentages are based on studies with Asian Americans and Other Racial Groups. ^c^ The three studies in this category had a racial group called “others” in which participants’ races were not explicitly named.

**Table 2:** Sample Size, Sex, Generational Statuses, and SESs of Asian Americans in 25 Reviewed Samples.

	*n* (%)	Article
Sample Size		
<50	5 (20%)	Lane et al.’s [19] Parkistani Sample, [22,33,36,37]
51–100	6 (24%)	Lane et al.’s [19] Indian Sample, Lane et al.’s [19] Bangladesh Sample, [7,27,30,39]
101–500	6 (24%)	Park’s [20] Chinese Sample, [21,23,29,34,35]
501–1000	2 (8%)	Park’s [20] Filipino Sample, [31]
>1001	6 (24%)	[24,25,26,28,32,38]
College Students or Not		
Non-College-Student Young Adults	4 (16%)	[20,23,25,35]
College Students	21 (84%)	All other samples
Sex		
Majority as Females	14 (56%)	Lane et al. [19] Samples 1–3, [21,22,23] *, [24,26,27,28,29,31,34,35]
Majority as Males	3 (12%)	Park’s [20] Chinese and Filipino Samples, [25]
Even Distribution of Females and Males	5 (20%)	[6,32,33,36,39]
Unknown	3 (12%)	[30,37,38]
Generational Status		
Roughly Even Distribution of 1st & 2nd	2 (8%)	[22,29]
Most 1st Generation	2 (8%)	Lane et al.’s [19] Parkistani Sample and Bangladesh Sample
Most 2nd Generation or Beyond	2 (8%)	Lane et al.’s [19] Indian Sample, [26]
Unknown	19 (76%)	All other samples
SES		
Mostly Middle Class	1 (4%)	[36]
Majority with Moderate Levels of Financial Stress	1 (4%)	[34]
Other (Including Unknown or Undetermined)	23 (92%)	all other samples

Note: Numbers used to denote each article correspond to the number in the reference list. * All female.

**Table 3:** Research Methods in 22 Reviewed Studies.

	*n* (%)	Article
Region of Data		
Regional Data	10 (45%)	[6,19,22,29,31,33,34,36,37,39]
National Data	12 (55%)	[20,21,23,24,25,26,27,28,32], [35] *, [38] *
Duration of Data Collection		
Longitudinal Data	3 (14%)	[6,20,25]
Cross-sectional Data	19 (86%)	All other studies
Method of Data Collection and Analysis		
Qualitative Method	2 (9%)	[22,27]
Mixed Method	2 (9%)	[29,34]
Quantitative Method	18 (82%)	All other studies
Sampling Methods		
Convenience Sampling	14 (64%)	[6,21,22,29,31,32,33,34,35,36,37,39]
Random Sampling	6 (27%)	[20,24,25,26,28,38]
Both Random and Purposive Sampling	2 (9%)	[27,30]

Note: Due to rounding, the total percentage for one variable sometimes is not 100%; numbers used to denote each article correspond to the number in the reference list. * These two studies collected data from online samples that were not necessarily representative of the population in the United States.

**Table 4:** Focused Suicide Outcomes in 22 Reviewed Studies.

	*n* (%)	Article
Variable Dimension		
Recent ^a^ Suicidal Ideation	13 (59%)	[6,19,20,23,25,27,28,29,30,32,34,35,39]
Lifetime Suicidal Ideation	1 (5%)	[38]
Recent ^a^ Suicide Attempts	2 (9%)	[31,32]
Lifetime Suicide Attempts	3 (14%)	[31,37,38]
Recent ^a^ Suicidality (Composite of Suicidal Ideation and Attempts)	1 (5%)	[24]
Past ^b^ Suicidality (Composite of Suicidal Ideation, Gestures, and Attempts)	1 (5%)	[22]
Recent ^a^ and Lifetime Suicidality (Composite of Suicidal Ideation and Attempts)	4 (18%)	[21,26,33,36]
Variable Type		
Continuous ^c^	11 (50%)	[6,19,21,26,29,30,33,34,35,36,39]
Dichotomous	11 (50%)	[20,22,23,24,25,27,28,31,32,37,38]

Note. The sum of the percentages was above 100% because nine studies had more than one suicide outcome; numbers used to denote each article correspond to the number in the reference list. ^a^ In Wong, Koo, et al. [29], “recent” refers to the past week; In Keum [35] and Keum et al. [23], “recent” refers to the past two weeks; In Yu & Chang [39], “recent” refers to the past month; in all other studies, “recent” refers to the past 12 months. ^b^ In Chung’s [22] study, “past” means during the college years. ^c^ Some of the continuous measures (e.g., the Suicide Behaviors Questionnaire-Revises) tap into additional dimensions of suicidality, such as threats of suicide and future likelihood of suicide.

**Table 5:** Risk and Protective Factors of STBs among AAYAs.

	Risk Factor & Article	Protective Factor & Article
Individual Level	**Mental health problems**: [28]	**Having personal reasons for living**: [21,27,34]
	**Depressive symptoms**: [20,26,31]	Self-reliance: [27]
	**Anxiety:** [34]	**Fear of suicide**: [27]
	**Hopelessness**: [19,33,36]	**Optimism**: [39]
	Loneliness: [26]	Sufficient sleep: [26,38]
	**Low self-esteem:** [30]	Physical activities: [26,38]
	**Generic state shame:** [30]	Independent self-construal: [29]
	Computer recreation use: [26]	Interdependent self-construal: [29]
	**Chemical use**: [26]	
	**Perfectionism**: [6]	
	**Low self-worth**: [22,29]	
	**Inadequate coping skills**: [22]	
	**Poor social problem-solving skills**: [6]	
	**Perceived burdensomeness**: [29,30,35]	
	**Thwarted belongingness**: [29]	
	Academic performance: [22,24,28,29,38]	
	Weight problems: [38]	
	**Sleep issues**: [38]	
	**Being female**: [38]	
	**Sexual orientation**: [24]	
	**Financial problems**: [28]	
	Food insecurity: [26]	
Relationship Level	**Conflict with family members**: [37]	**Social support**: [27]
	**Family problems**: [28]	**Socializing**: [26]
	Poor quality of romantic relationships: [22]	**Family responsibilities**: [26]
	Conflict with romantic partners: [37]	Living with family members or roommates: [28]
	Conflict with peers or friends: [37]	
	Interpersonal Shame: [30]	
Community Level	Verbal threats on campus: [24]	Religious affiliations: [28]
	Involvement in student organizations: [28]	Transitions to college life: [22]
Societal Level	Public stigma about mental health: [32]	None
	Acculturation: [25] Cultural conflict: [29] Acculturative stress: [31]	Desire not to burden others: [27]
	Family shame: [30]	
	Racial/ethnic discrimination: [26,29,31]	
Cultural	Gender discrimination: [29]	
Level	Gendered racial microaggressions: [23]	
	Online racism: [35]	
	Internalized racism: [23]	
	Online racism: [35]	
	Internalized racism: [23]	

Note: Bold font indicates that the factor was also reviewed in Cramer and Kapusta’s [14] study; numbers used to denote each article correspond to the number in the reference list.

## Data Availability

No new data were created or analyzed in this study. Data sharing is not applicable to this article.

## References

[1] Institute of Medicine and National Research Council (2015). Investing in the Health and Well-Being of Young Adults.

[2] Franklin J.C., Ribeiro J.D., Fox K.R., Bentley K.H., Kleiman E.M., Huang X., Musacchio K.M., Jaroszewski A.C., Chang B.P., Nock M.K. (2017). Risk factors for suicidal thoughts and behaviors: A meta-analysis of 50 years of research. Psychol. Bull..

[3] Centers for Disease Control and Prevention (2020). WISQARS Leading Causes of Death Reports. https://wisqars.cdc.gov/fatal-leading.

[4] Substance Abuse and Mental Health Services Administration (2023). 2021 National Survey on Drug Use and Health: Among the Asian Population Aged 12 or Older. https://www.samhsa.gov/data/release/2021-national-survey-drug-use-and-health-nsduh-releases.

[5] Brener N.D., Hassan S.S., Barrios L.C. (1999). Suicidal ideation among college students in the United States. J. Consult. Clin. Psychol..

[6] Chang E.C. (1998). Cultural differences, perfectionism, and suicidal risk in a college population: Does social problem solving still matter?. Cogn. Ther. Res..

[7] Liu C.H., Stevens C., Wong S.H.M., Yasui M., Chen J.A. (2019). The prevalence and predictors of mental health diagnoses and suicide among U.S. college students: Implications for addressing disparities in service use. Depress. Anxiety.

[8] Martell R.M., King M. (2021). The Rate of Student Death from Suicide from the Big Ten Counseling Centers: 2009–2018. Mental Health, Substance Use, and Wellbeing in Higher Education: Supporting the Whole Student.

[9] Masten A.S. (2001). Ordinary magic: Resilience processes in development. Am. Psychol..

[10] Marraccini M.E., Griffin D., O’Neill J.C., Martinez R.R., Chin A.J., Toole E.N., Grapin S.L., Naser S.C. (2022). School Risk and Protective Factors of Suicide: A Cultural Model of Suicide Risk and Protective Factors in Schools. Sch. Psychol. Rev..

[11] Wyatt L.C., Ung T., Park R., Kwon S.C., Trinh-Shevrin C. (2015). Risk factors of suicide and depression among Asian American, Native Hawaiian, and Pacific Islander youth: A systematic literature review. J. Health Care Poor Underserved.

[12] Cramer R.J., Judah M.R., Badger N.L., Holley A.M., Judd S., Peterson M., Hager N., Vandecar-Burdin T., Foss J.J. (2022). Suicide on college campuses: A public health framework and case illustration. J. Am. Coll. Health.

[13] McLaren L., Hawe P. (2005). Ecological perspectives in health research. J. Epidemiol. Community Health.

[14] Cramer R.J., Kapusta N.D. (2017). A Social-Ecological Framework of Theory, Assessment, and Prevention of Suicide. Front. Psychol..

[15] Chu J., Goldblum P., Floyd R., Bongar B. (2010). The cultural theory and model of suicide. Appl. Prev. Psychol..

[16] Chu J., Floyd R., Diep H., Pardo S., Goldblum P., Bongar B. (2013). A tool for the culturally competent assessment of suicide: The Cultural Assessment of Risk for Suicide (CARS) measure. Psychol. Assess..

[17] Chu J., Maruyama B., Batchelder H., Goldblum P., Bongar B., Wickham R.E. (2020). Cultural pathways for suicidal ideation and behaviors. Cult. Divers. Ethn. Minor. Psychol..

[18] Moher D., Shamseer L., Clarke M., Ghersi D., Liberati A., Petticrew M., Shekelle P., Stewart L.A., PRISMA-P Group (2015). Preferred Reporting Items for Systematic Review and Meta-Analysis Protocols (PRISMA-P) 2015 statement. Syst. Rev..

[19] Lane R., Cheref S., Miranda R. (2016). Ethnic differences in suicidal ideation and its correlates among South Asian American emerging adults. Asian Am. J. Psychol..

[20] Park S.-Y. (2017). Depressive Symptoms and Suicidal Ideation from Adolescence to Young Adulthood in Chinese American and Filipino American Youth. J. Soc. Soc. Work Res..

[21] Choi J.L., Rogers J.R. (2010). Exploring the validity of the College Student Reasons for Living Inventory among Asian American college students. Arch. Suicide Res..

[22] Chung I.W. (2003). Examining suicidal behavior of Asian American female college students: Implications for practice. J. Coll. Stud. Psychother..

[23] Keum B.T., Wong M.J., Salim-Eissa R. (2023). Gendered racial microaggressions, internalized racism, and suicidal ideation among emerging adult Asian American women. Int. J. Soc. Psychiatry.

[24] Maffini C.S. (2018). Campus safety experiences of Asian American and Asian international college students. Asian Am. J. Psychol..

[25] Park S.-Y., Park S.-Y. (2020). Immigration and language factors related to depressive symptoms and suicidal ideation in Asian American adolescents and young adults. Community Ment. Health J..

[26] Rivera Juarez A.G., Prichard J.R., Berg S.S. (2023). Psychological Well-Being in Asian and Asian American University Students: Impacts of Discrimination During the COVID-19 Pandemic. J. Adolesc. Health Off. Publ. Soc. Adolesc. Med..

[27] Tran K.K., Wong Y.J., Cokley K.O., Brownson C., Drum D., Awad G., Wang M.C. (2015). Suicidal Asian American college students’ perceptions of protective factors: A qualitative study. Death Stud..

[28] Wong Y.J., Brownson C., Schwing A.E. (2011). Risk and protective factors associated with Asian American students’ suicidal ideation: A multicampus, national study. J. Coll. Stud. Dev..

[29] Wong Y.J., Koo K., Tran K.K., Chiu Y.C., Mok Y. (2011). Asian American college students’ suicidal ideation: A mixed-methods study. J. Couns. Psychol..

[30] Wong Y.J., Kim B.S., Nguyen C.P., Cheng J.K.Y., Saw A. (2014). The interpersonal shame inventory for Asian Americans: Scale development and psychometric properties. J. Couns. Psychol..

[31] Gomez J., Miranda R., Polanco L. (2011). Acculturative stress, perceived discrimination, and vulnerability to suicide attempts among emerging adults. J. Youth Adolesc..

[32] Goodwill J.R., Zhou S. (2020). Association between perceived public stigma and suicidal behaviors among college students of color in the US. J. Affect. Disord..

[33] Hirsch J.K., Visser P.L., Chang E.C., Jeglic E.L. (2012). Race and ethnic differences in hope and hopelessness as moderators of the association between depressive symptoms and suicidal behavior. J. Am. Coll. Health.

[34] Hwang W.C., Goto S. (2008). The impact of perceived racial discrimination on the mental health of Asian American and Latino college students. Cult. Divers. Ethn. Minor. Psychol..

[35] Keum B.T. (2023). Impact of Online Racism on Suicide Ideation Through Interpersonal Factors Among Racial Minority Emerging Adults: The Role of Perceived Burdensomeness and Thwarted Belongingness. J. Interpers. Violence.

[36] Muehlenkamp J.J., Gutierrez P.M., Osman A., Barrios F.X. (2005). Validation of the Positive and Negative Suicidal ideation (PANSI) Inventory in a diverse sample of young adults. J. Clin. Psychol..

[37] Rosario-Williams B., Rowe-Harriott S., Ray M., Jeglic E., Miranda R. (2022). Factors precipitating suicide attempts vary across race. J. Am. Coll. Health.

[38] Sa J., Choe C.S., Cho C.B.Y., Chaput J.P., Lee J., Hwang S. (2020). Sex and racial/ethnic differences in suicidal consideration and suicide attempts among US college students, 2011–2015. Am. J. Health Behav..

[39] Yu E.A., Chang E.C. (2016). Optimism/pessimism and future orientation as predictors of suicidal ideation: Are there ethnic differences?. Cult. Divers. Ethn. Minor. Psychol..

[40] Osman A., Bagge C.L., Gutierrez P.M., Konick L.C., Kopper B.A., Barrios F.X. (2001). The Suicidal Behaviors Questionnaire-Revised (SBQ-R): Validation with clinical and nonclinical samples. Assessment.

[41] Van Orden K.A., Witte T.K., Cukrowicz K.C., Braithwaite S., Selby E.A., Joiner T.E. (2010). The Interpersonal Theory of Suicide. Psychol. Rev..

[42] Yee B.W.K., DeBaryshe B.D., Yuen S., Kim S.Y., McCubbin H.I., Leong F.T.L., Ebreo A., Kinoshita L., Inman A.G., Yang L.H., Fu M. (2007). Asian American and Pacific Islander families: Resiliency and life-span socialization in a cultural context. Handbook of Asian American Psychology.

[43] Campbell F., Blank L., Cantrell A., Baxter S., Blackmore C., Dixon J., Goyder E. (2022). Factors that influence mental health of university and college students in the UK: A systematic review. BMC Public Health.

[44] Sheldon E., Simmonds-Buckley M., Bone C., Mascarenhas T., Chan N., Wincott M., Gleeson H., Sow K., Hind D., Barkham M. (2021). Prevalence and risk factors for mental health problems in university undergraduate students: A systematic review with meta-analysis. J. Affect. Disord..

[45] Lai D.W.L., Li L., Daoust G.D. (2017). Factors Influencing Suicide Behaviours in Immigrant and Ethno-Cultural Minority Groups: A Systematic Review. J. Immigr. Minor. Health.

[46] Siddiqui S.M. (2022). Acculturative stress, everyday racism, and mental health among a community sample of South Asians in Texas. Front. Public Health.

[47] Huang X., Fan B. (2024). The Association Between Campus Climate and the Mental Health of LGBTQ+ College Students: A Systematic Review and Meta-Analysis. Sex. Cult..

[48] Tierney W.G. (1999). Models of Minority College-Going and Retention: Cultural Integrity versus Cultural Suicide. J. Negro Educ..

[49] Lee E., McGoldrick M., Giordano J., Pearce J. (1996). Chinese Families. Ethnicity and Family Therapy.

[50] Klonsky E.D., May A.M., Saffer B.Y. (2016). Suicide, Suicide Attempts, and Suicidal Ideation. Annu. Rev. Clin. Psychol..

[51] Li W., Dorstyn D.S., Denson L.A. (2016). Predictors of Mental Health Service Use by Young Adults: A Systematic Review. Psychiatr. Serv..

[52] Goodwin J., Savage E., Horgan A. (2016). Adolescents’ and Young Adults’ Beliefs about Mental Health Services and Care: A Systematic Review. Arch. Psychiatr. Nurs..

[53] Aguey-Zinsou M., Scanlan J.N., Cusick A. (2022). A Scoping and Systematic Review of Employment Processes and Outcomes for Young Adults Experiencing Psychosis. Community Ment. Health J..

[54] Smajlović D. (2015). Strokes in young adults: Epidemiology and prevention. Vasc. Health Risk Manag..

[55] Arnett J.J. (2015). Emerging Adulthood: The Winding Road from the Late Teens Through the Twenties.

